# Electropolymerization on wireless electrodes towards conducting polymer microfibre networks

**DOI:** 10.1038/ncomms10404

**Published:** 2016-01-25

**Authors:** Yuki Koizumi, Naoki Shida, Masato Ohira, Hiroki Nishiyama, Ikuyoshi Tomita, Shinsuke Inagi

**Affiliations:** 1 Department of Electronic Chemistry, Interdisciplinary Graduate School of Science and Engineering, Tokyo Institute of Technology, 4259 Nagatsuta-cho, Midori-ku, Yokohama 226-8502, Japan

## Abstract

Conducting polymers can be easily obtained by electrochemical oxidation of aromatic monomers on an electrode surface as a film state. To prepare conducting polymer fibres by electropolymerization, templates such as porous membranes are necessary in the conventional methods. Here we report the electropolymerization of 3,4-ethylenedioxythiophene and its derivatives by alternating current (AC)-bipolar electrolysis. Poly(3,4-ethylenedioxythiophene) (PEDOT) derivatives were found to propagate as a fibre form from the ends of Au wires used as bipolar electrodes (BPEs) parallel to an external electric field, without the use of templates. The effects of applied frequency and of the solvent on the morphology, growth rate and degree of branching of these PEDOT fibres were investigated. In addition, a chain-growth model for the formation of conductive material networks was also demonstrated.

## Abstract (web summary)

Electropolymerization of aromatic monomers on bipolar electrodes is emerging as promising route to the surface modification of conductive objects. Here, the authors discover that some conducting polymers propagate as fibres, opening up the possibility of growing conductive polymer networks via a wireless process.

## Graphical abstract

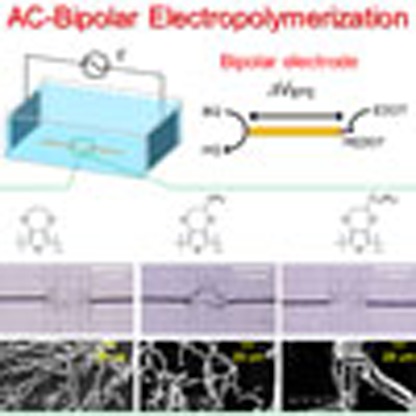

Recently, there have been a number of interesting reports concerning bipolar electrochemistry, in which anodic and cathodic reactions take place simultaneously on both poles of a conductive material placed between a pair of driving electrodes^1^,^2^,^3^,^4^,^5^,^6^,^7^,^8^,^9^,^10^,^11^,^12^. The functioning of this special wireless electrode (or bipolar electrode (BPE)) is induced by an external electric field generated in a low concentration of a supporting electrolyte. Considering the potential energy diagram under the application of a direct current (DC) voltage (*E*) between the driving electrodes, the potential of BPEs is floating to an equilibrium value (*E*_elec_) in a gradient of solution potential, consequently the anodic and cathodic overpotentials (*ΔV*_BPE_) can drive redox reactions at each BPE in the same manner (Fig. 1)^3^,^5^. In one of the most significant studies, Kuhn *et al*. demonstrated the elegant surface modification of conductive particles^13^,^14^,^15^,^16^, involving metal plating (cathodic reduction of metal ions) at one pole in conjunction with the electrochemical oxidation (electropolymerization) of pyrrole at the opposite pole to give Janus-type particles coated simultaneously with metal and a conducting polymer film (polypyrrole)^13^.

Our approach in obtaining bifunctional particles, such as these, has been to employ alternating current (AC)-bipolar electrochemistry, through which a variety of glassy carbon (GC) particles modified with gold in a site selective manner have been synthesized^17^. The next challenge regarding this technology was to demonstrate the iterative electropolymerization of aromatic monomers on both poles of a BPE using this AC-bipolar system, and preliminary work using pyrrole as a monomer gave GC particles modified with polypyrrole films, as expected. However, to our surprise, the AC-bipolar electrolysis of 3,4-ethylenedioxythiophene (EDOT) resulted in the formation of polymer fibres at both poles of the GC-BPE, rather than film deposition. To the best of our knowledge, the propagation of conducting polymer microfibres from the very edges of wireless electrodes parallel to the external electric field and subsequent network formation have not previously been observed. So far, fibre/wire-like conducting polymers have been prepared by the conventional electropolymerization using templates such as porous membranes or seeds^18^,^19^,^20^,^21^. In another system of the electropolymerization of aromatic monomers with conventional ‘wired' working electrodes using an AC voltage, conducting polymer film was deposited from two working electrodes along with the two-/three-dimensional formation of dendrites and connected each other^22^,^23^. However, it was difficult to control finely the size of dendrites, thus the method for the interconnection of electrodes was not satisfactory towards practical applications. In this context, this spontaneous propagation of conducting polymer microfibres from wireless electrodes is thus a new phenomenon and is worthy of further, detailed investigation.

## Results

### AC-bipolar electropolymerization

**Figure 1:**
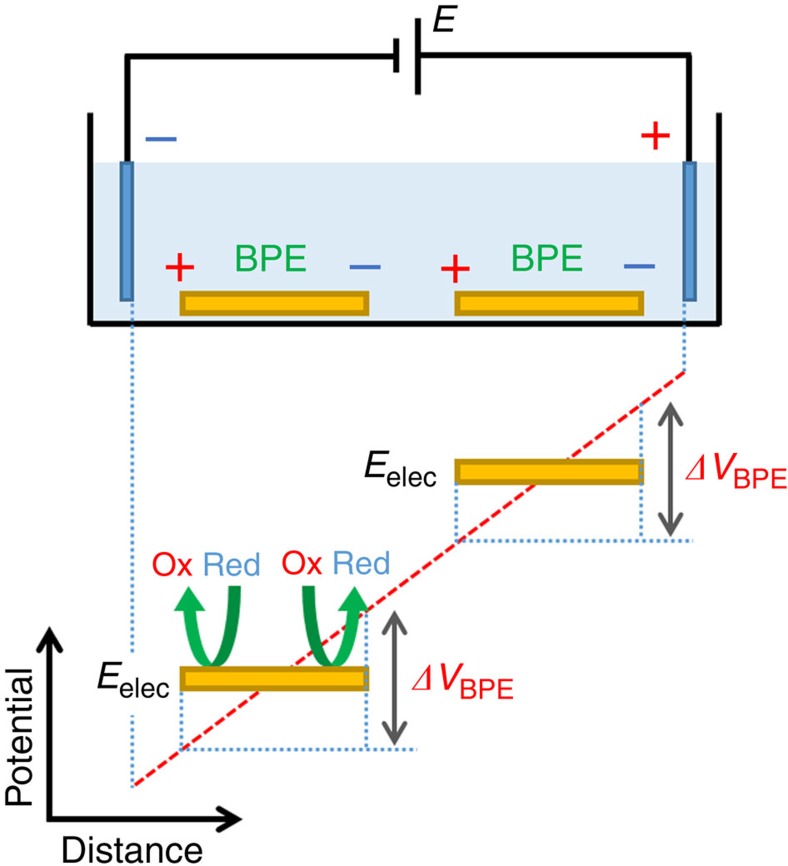
Schematic illustration of the principle of bipolar electrochemistry. The potential difference at the electrode/solution interface varies across the length of BPEs according to the potential gradient applied to the solution; thus, the overpotentials (*ΔV*_BPE_) can drive redox reactions at both sides.

**Figure 2:**
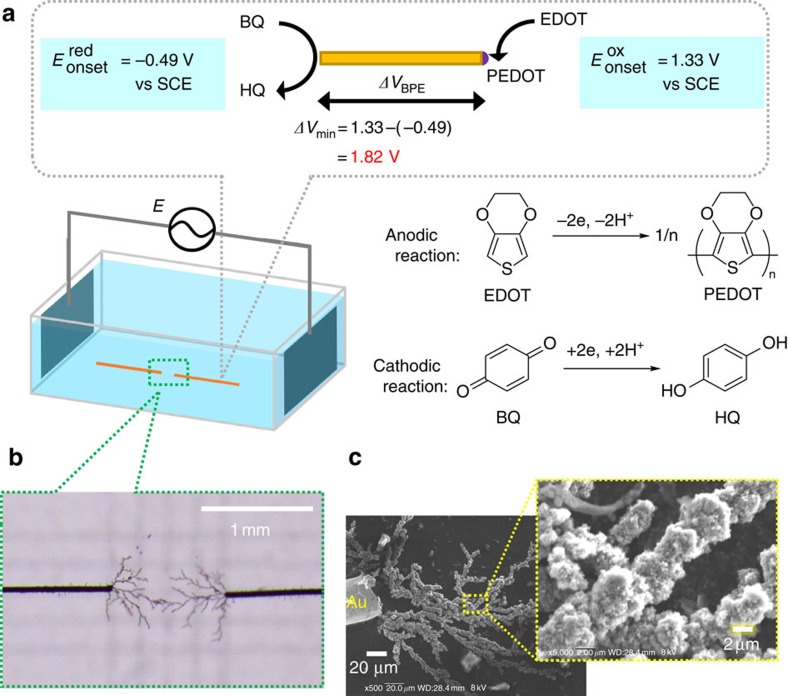
AC-bipolar electropolymerization of EDOT. (**a**) Schematic representation of the electrochemical setup for AC-bipolar electrolysis including oxidative polymerization of EDOT and sacrificial reduction of BQ with Au wires (*φ*=50 μm, 20 mm) as BPEs set in between Pt driving electrodes (20 × 20 mm, distance: 60 mm), (**b**) optical microscope image of PEDOT fibres bridging the 1 mm gap between Au wires (*ΔV*_BPE_=8.3 V, 90 s) and (**c**) SEM images of the PEDOT fibres. HQ, hydroquinone; SCE, saturated calomel electrode.

To assess the reaction sites on the BPE, gold (Au) wires (*φ*=50 μm, length=20 mm) placed 1 mm apart from one another were employed as BPEs and the reaction process was monitored using an optical microscope. The experimental configuration is summarized in Fig. 2a, consisting of a pair of platinum feeder electrodes (20 × 20 mm, distance: 60 mm) in 1 mM tetrabutylammonium perchlorate (Bu_4_NClO_4_)/acetonitrile (MeCN) containing 50 mM EDOT monomer and 5 mM benzoquinone (BQ) as a sacrificial reagent for reduction. During the functioning of the Au wires as BPEs, the electropolymerization of EDOT takes place at the anodic part of the Au wire, while the sacrificial reduction of BQ to hydroquinone simultaneously proceeds at the cathodic part of the wire (Supplementary Fig. 1). Based on the principles of bipolar electrochemistry (Fig. 1), the potential difference applied across a BPE (*ΔV*_BPE_) can be estimated from the voltage between the driving electrodes (*E*) and the length of the wire, weighted by the cell factors (Supplementary Fig. 2 and Supplementary Equation 6)^17^. The redox reactions will proceed when *ΔV*_BPE_ is sufficiently above their potential difference value (*ΔV*_min_, Fig. 2a).

On the application of AC voltage (*E*=30 V, *ΔV*_BPE_=8.3 V, 5 Hz, square wave alternating in polarity) between the driving electrodes, several polymer fibres were observed to propagate dendritically from the end of each Au wire. The resulting iterative AC-bipolar electropolymerization of EDOT generated poly(3,4-ethylenedioxythiophene) (PEDOT) fibres. The real-time observation of the gradual propagation of the PEDOT fibres revealed that the terminals of the growing fibres were activated for further electropolymerization (Supplementary Movie). After 90 s, the tip of one of the propagating fibres met the tip of a fibre growing from the other Au wire and the fibres connected to one another, bridging the 1 mm gap between the Au wires (Fig. 2b). Following this, the propagation of the fibres abruptly ceased, because the two Au wires were now connected by the conducting polymer fibres and hence behaved as a single BPE. Providing further evidence of this, the other ends of the Au wires, which were closer to the feeder electrodes, still worked as anodic and cathodic poles for the electropolymerization of EDOT, and further propagation of fibres was observed (Supplementary Fig. 3). During the bipolar electropolymerization experiment, PEDOT was not produced on the driving electrodes. Although the oxidation of EDOT should occur at the driving anode, the potential applied was probably too low (most of voltage applied was lost due to the potential drop in the solution) to conduct the effective electropolymerization.

When a smaller voltage (*E*=21 V, *ΔV*_BPE_=5.8 V) was applied, the propagation rate of the PEDOT fibre was apparently too slow to bridge the Au wires (Supplementary Fig. 4). However, under such moderate conditions, the degree of branching was also small. In contrast, the application of a larger voltage (*E*=39 V, *ΔV*_BPE_=10.8 V) resulted in similar propagation rate to that with *ΔV*_BPE_=8.3 V because the fibre formation process at the high *ΔV*_BPE_ application was diffusion-limited.

The PEDOT fibres and the connected Au wires were successfully transferred onto a carbon tape after carefully washing with MeCN and drying, and Fig. 2c shows scanning electron microscopy (SEM) images of the fibres. It was found that each fibre was composed of clusters connected in a linear fashion and had a diameter of ∼3–5 μm. Energy dispersive X-ray mapping of the same observation area indicated the presence of sulfur derived from the EDOT moiety and generated an image in good agreement with the SEM image of the fibres (Supplementary Fig. 5). Such fibre propagation with a uniform thickness is totally different from the case of the conventional AC-electropolymerization giving the irregular conducting polymer dendrites^22^,^23^.

### Fibre-propagation mechanism

**Figure 3:**
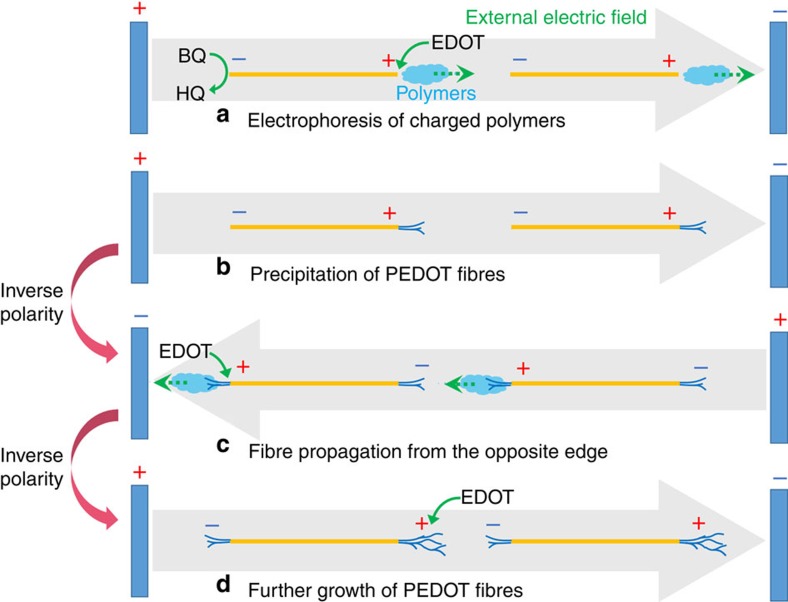
Proposed PEDOT fibre-propagation mechanism. (**a**) Electrophoresis of charged polymers, (**b**) precipitation of PEDOT fibres, (**c**) propagation of fibres from the opposite end and (**d**) further growth of PEDOT fibres.

Here we propose a possible propagation mechanism by which the PEDOT fibres are grown through AC-bipolar electropolymerization (Fig. 3). Initially, the external electric field generates Au-BPEs, at which oxidation of the EDOT monomer and reduction of BQ take place (Fig. 3a). The former results in polymerization of EDOT and, once the polymer has grown sufficiently, it becomes insoluble and deposits on one end of the Au wire. It is well known that the oxidation potential of PEDOT is less positive than that of EDOT monomer. During polymerization of EDOT under bipolar electrochemical conditions, the resulting polymer is typically doped and has cationic charges, and so can be electrophoresed under the influence of the external electric field. Consequently, the polymer is deposited not as a film but rather in an anisotropic morphology (Fig. 3b). Under a contrary electric field, Au-BPEs with opposite polarities are generated and similar electrode reactions take place (Fig. 3c). Upon repeated cycling of the AC power supply, the edges of the PEDOT fibres, where the *ΔV*_BPE_ is the highest, grow in a manner parallel to the external electric field, serving as active sites for the electropolymerization because of their sufficiently high conductivity (Fig. 3d). Accordingly, the electrophoresis of the charged polymer species plays an important role in the fibre-propagation process. In a previous report on the copper wire formation between copper particles by the DC-bipolar electrolysis, the electrophoresis of copper ion in a low concentration of electrolyte was necessary to determine the propagation direction^24^.

We next examined the AC-bipolar electropolymerization of pyrrole and thiophene as monomers; however, these did not give fibres but rather formed films covering the BPEs (Supplementary Fig. 6). This seems to be explained by the inactivation of the BPEs covered with the relatively low electric conductivity of these polymers compared to that of PEDOT. It should be noted that, under a DC voltage, there appeared no PEDOT fibre at the ends of the Au wires, while the migration of blue-coloured polymers parallel to the external electric field was observed. Alternatively, PEDOT films and clusters were continuously deposited on the anodic portion of the wire (Supplementary Fig. 7a). In the cases of pyrrole and thiophene under a DC voltage, the corresponding polymer films were formed but more slowly and thinly than that of PEDOT probably owing to the low conductivity of the polymers (Supplementary Fig. 7b,c). This supports the result of the unsuccessful fibre formation under AC-bipolar electropolymerization mentioned above.

When a higher frequency of 50 Hz for AC voltage was applied, the number of generated PEDOT fibres was increased and highly connected networks were obtained (Supplementary Fig. 8). The diameter of the resulting fibres was decreased to ∼1–2 μm compared with that of the fibres prepared using a lower frequency of 5 Hz as evidenced by the SEM observation (Supplementary Fig. 9). Considering the propagation mechanism described in Fig. 3, the frequency is evidently an important factor determining fibre morphology. The main difference between the frequencies is in the diffusion length of the charged polymers during electrophoresis at each anodic moment. When applying a lower frequency, the diffusion length of the charged polymers is relatively long, and the local concentration of the active species for polymerization is lower. In contrast, a higher local concentration of the charged polymers is expected at higher frequencies. This model explains why the quantity of PEDOT fibres increased when applying a higher frequency. The difference in the diffusion length of the polymer also affects propagation rate of fibres. The lower frequency (5 Hz) accelerated it. In addition, the application of a much higher frequency (100 Hz) ended in failure of forming of any fibres under the conditions. Since it takes longer time to form electric double layers in low concentration of an electrolyte, such a higher frequency was not suitable for the AC-bipolar electropolymerization. The supporting salts were also found to affect the morphology and propagation rate of the fibres, as shown in Supplementary Fig. 9, with the complicated factors as follows. In general, counter ions, which compensate for the cationic state of PEDOT, play a crucial role in its polymerization rate and morphology^25^,^26^. Ionic conductivity should be related to the rate of the double layer formation. The solubility of the growing PEDOT seems to be another important factor in determining the size and the morphology of the fibres; the use of dichloromethane (CH_2_Cl_2_) in place of MeCN reduced the propagation rate such that a span of 540 s was required to bridge the Au wires (Supplementary Fig. 10).

### Scope of EDOT monomers

**Figure 4:**
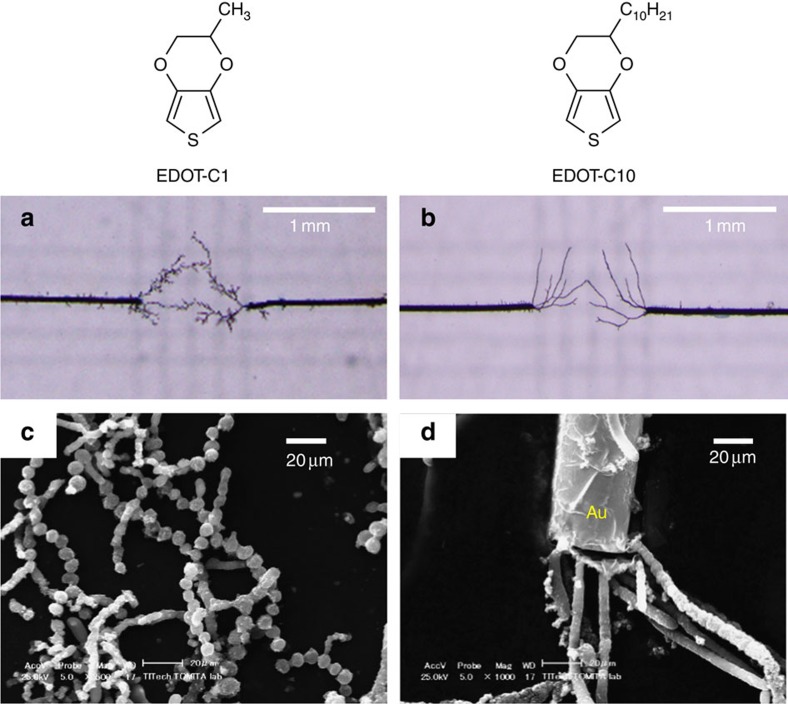
Versatile fibre morphology of PEDOT derivatives. (**a**,**b**) Optical microscope and (**c**,**d**) SEM images of the polymer fibres obtained using EDOT-C1 (**a**,**c**) and EDOT-C10 (**b**,**d**) as monomers.

To investigate the scope of the AC-bipolar electropolymerization, two additional EDOT derivatives were also assessed (Fig. 4). In the case of the EDOT-C1 monomer, rosary-like PEDOT fibres were obtained with a smooth surface, while the polymerization of EDOT-C10 resulted in the formation of rod-like fibres. In both cases, the degree of branching of the fibres was decreased compared with the extent observed for EDOT. It was therefore possible to create versatile fibre structures using EDOT derivatives, although the relationship between the chemical structure of the monomer and the morphology of the resulting PEDOT fibres is still unclear at the present time.

### Selective network formation

Towards the practical connection of an Au wire intersection with the PEDOT fibres, we prepared the setup under the application of electric fields with the different direction as shown in Supplementary Fig. 11. In the case of Supplementary Fig. 11a, only two Au wires put parallel to the electric field were active as BPEs and connected each other, while the other two Au wires were inactive to promote redox reactions because of their insufficient length crossing the electric field. On the other hand, the case of Supplementary Fig. 11b resulted in the successful interconnection of the Au wires in the different mode in accordance with the direction of the electric field. The conductive networks of the Au wires and the PEDOT fibres were achieved selectively.

**Figure 5:**
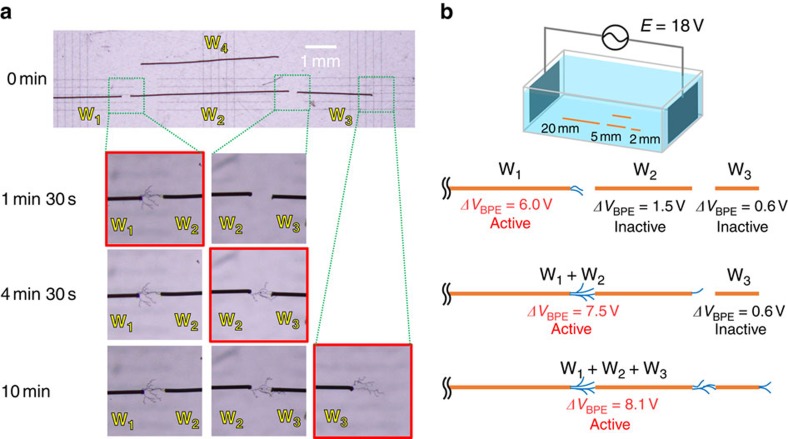
Chain-growth model for the formation of conductive material networks. (**a**) Optical microscope images of Au wires (W_1_: 20 mm, W_2_: 5 mm, W_3_: 2 mm and W_4_: 4.5 mm) on the chain-growth model experiment by the AC-bipolar electropolymerization of EDOT. (**b**) Illustration showing the proposed mechanism of the chain-growth model.

From the foregoing findings, it seems possible to sequentially activate conductors with different lengths (that is, different *ΔV*_BPE_) by the connection with PEDOT fibres once grown from an active BPE. Finally, we demonstrated the ‘chain-growth model' for the formation of conducting networks using the bipolar electrochemical method. As shown in Fig. 5, three kinds of Au wires (W_1_–W_3_, with lengths of 20, 5 and 2 mm) were placed between the driving electrodes, 0.3 mm apart from one another. During the application of AC voltage (*E*=18 V, 5 Hz) between the driving electrodes, the value of *ΔV*_BPE_ for these wires was 6.0, 1.5 and 0.6 V, respectively; thus, only W_1_ was active as a BPE for the electropolymerization of EDOT. PEDOT, therefore, propagated from the end of W_1_ and contacted the end of W_2_ in a span of 90 s. W_2_ then became active to initiate the propagation of PEDOT from its right end owing to the sufficient *ΔV*_BPE_ value (7.5 V) of the combined BPE (W_1_ and W_2_), with the resulting fibre reaching W_3_ after 270 s. Finally the right end of W_3_ became an active site for further electropolymerization. Throughout the experiment, an additional wire (W_4_) with a length of 4.5 mm acting as a reference remained intact since it received an electric field insufficient to drive the polymerization. This model demonstrated the one-directional propagation of a conducting network of metal wires and PEDOT fibres from an initiator by an electrochemical stimulus, taking advantages of the bipolar electrochemistry.

## Discussion

We have successfully demonstrated the unusual electropolymerization behaviour of the EDOT monomer, leading to one-dimensional propagation from both ends of Au wires acting as BPEs without direct feeding of an electric potential under AC-bipolar electrochemical conditions. The electrophoresis of charged polymers evidently played an important role in obtaining various PEDOT microfibre structures. The morphology of the fibres, as well as the propagation rate and degree of branching were found to be dependent on the frequency, solvent and supporting electrolyte used. This spontaneous propagation of conducting polymer fibres in the absence of templates should be widely applicable to the creation of conductive material networks with a wireless process. The chain-growth model we demonstrated is a totally novel mode to activate a small conductor as a BPE under the application of a mild electric field.

## Methods

### Materials

All reagents and chemicals were obtained from commercial sources and used without further purification otherwise noted. EDOT-C10 was prepared according to a procedure defined in the literature^27^. Gold (Au) wires and platinum (Pt) plates were purchased from commercial sources.

### Synthesis of EDOT-C1

EDOT-C1 was synthesized via transetherification similarly to the literature procedure^27^ using 1,2-propanediol as a diol. Yield: 72%, yellowish oil. ^1^H NMR (270 MHz, CDCl_3_): δ 6.31 (s, 1H); 6.30 (s, 1H); 4.27 (m, 1H); 4.13 (dd, *J*=11.5 Hz, 2.1 Hz, 1H); 3.82 (dd, *J*=11.5 Hz, 8.5 Hz, 1H); and 1.34 (d, *J*=6.5 Hz, 3H). ^13^C NMR (68 MHz, CDCl_3_): δ 142.16, 141.41, 99.30, 99.29, 70.00, 69.43 and 16.24. High resolution MS (EI): *m*/*z* [M^+^] calculated for C_7_H_8_O_2_S_1_: 156.0245; found:156.0245.

### A typical procedure for AC-bipolar electropolymerization

The bipolar electrolysis apparatus shown in Fig. 2a was employed, containing an electrolytic solution of MeCN (1 mM), EDOT (50 mM) and BQ (5 mM). An AC voltage (*E*=30 V, 5 Hz) was applied between the driving electrodes for the desired time span at room temperature. Following electrolysis, the Au wires, now connected by PEDOT fibres, were carefully washed with solvent and dried.

### Measurements

DC and AC power were supplied to the driving electrodes using an EC1000SA AC/DC power source (NF Corporation). Optical microscope observations were conducted with an Olympus SZX10 and a Keyence VHX-5000, and SEM observations were performed using a Shimadzu SS-550. Energy dispersive X-ray spectra were acquired with a Keyence Genesis XM2 and cyclic voltammetry measurements were carried out using an ALS 6005C Electrochemical Analyzer.

## Additional information

**How to cite this article:** Koizumi, Y. *et al*. Electropolymerization on wireless electrodes toward conducting polymer microfibre networks. *Nat. Commun.* 7:10404 doi: 10.1038/ncomms10404 (2016).

## Supplementary Material

Supplementary Information Supplementary Figures 1-13, Supplementary Methods and Supplementary References

Supplementary Movie 1 Real-time optical microscope image of AC-bipolar electropolymerization.

## References

[b1] Loget G., Zigah D., Bouffler L., Sojic N. & Kuhn A. Bipolar electrochemistry: from materials science to motion and beyond. Acc. Chem. Res. 46, 2513–2523 (2013) . 23719628 10.1021/ar400039k

[b2] Fosdick S. E., Knust K. N., Scida K. & Crooks R. M. Bipolar electrochemistry. Angew. Chem. Int. Ed. 52, 10438–10456 (2013) . 10.1002/anie.201300947 23843205

[b3] Mavré F. . Bipolar electrodes: a useful tool for concentration, separation, and detection of analytes in microelectrochemical systems. Anal. Chem. 82, 8766–8774 (2010) . 20815405 10.1021/ac101262v

[b4] Inagi S. & Fuchigami T. Electrochemical post-functionalization of conducting polymers. Macromol. Rapid Commun. 35, 854–867 (2014) . 24590504 10.1002/marc.201400023

[b5] Chow K.-F., Mavré F., Crooks J. A., Chang B.-Y. & Crooks R. M. A large-scale, wireless electrochemical bipolar electrode microarray. J. Am. Chem. Soc. 131, 8364–8365 (2009) . 19530725 10.1021/ja902683f

[b6] Loget G. & Kuhn A. Propulsion of microobjects by dynamic bipolar self-regeneration. J. Am. Chem. Soc. 132, 15918–15919 (2010) . 20964295 10.1021/ja107644x

[b7] Loget G. & Kuhn A. Electric field-induced chemical locomotion of conducting objects. Nat. Commun. 2, 535 (2011) . 22086336 10.1038/ncomms1550

[b8] Ulrich C., Andersson O., Nyholm L. & Björefors F. Formation of molecular gradients on bipolar electrodes. Angew. Chem. Int. Ed. 47, 3034–3036 (2008) . 10.1002/anie.200705824 18327762

[b9] Inagi S., Ishiguro Y., Atobe M. & Fuchigami T. Bipolar patterning of conducting polymers by electrochemical doping and reaction. Angew. Chem. Int. Ed. 49, 10136–10139 (2010) . 10.1002/anie.201005671 21117114

[b10] Ishiguro Y., Inagi S. & Fuchigami T. Site-controlled application of electric potential on a conducting polymer “canvas”. J. Am. Chem. Soc. 134, 4034–4036 (2012) . 22353050 10.1021/ja211774z

[b11] Inagi S., Nagai H., Tomita I. & Fuchigami T. Parallel polymer reactions of a polyfluorene derivative by electrochemical oxidation and reduction. Angew. Chem. Int. Ed. 52, 6616–6616 (2013) . 10.1002/anie.201302251 23666748

[b12] Shida N., Koizumi Y., Nishiyama H., Tomita I. & Inagi S. Electrochemically mediated atom transfer radical polymerization from a substrate surface manipulated by bipolar electrolysis: Fabrication of gradient and patterned polymer brushes. Angew. Chem. Int. Ed. 54, 3922–3926 (2015) . 10.1002/anie.201412391 25704396

[b13] Loget G. . Versatile procedure for synthesis of Janus-type carbon tubes. Chem. Mater. 23, 2595–2599 (2011) .

[b14] Loget G. & Kuhn A. Bulk synthesis of Janus objects and asymmetric patchy particles. J. Mater. Chem. 22, 15457–15474 (2012) .

[b15] Loget G., Roche J. & Kuhn A. True bulk synthesis of Janus objects by bipolar electrochemistry. Adv. Mater. 24, 5111–5116 (2012) . 22806760 10.1002/adma.201201623

[b16] Ongaro M., Gambirasi A., Favaro M., Kuhn A. & Ugo P. Asymmetrical modification of carbon microfibers by bipolar electrochemistry in acetonitrile. Electrochim. Acta 116, 421–428 (2014) .

[b17] Koizumi Y., Shida N., Tomita I. & Inagi S. Bifunctional modification of conductive particles by iterative bipolar electrodeposition of metals. Chem. Lett. 43, 1245–1247 (2014) .

[b18] Martin C. R. Nanomaterials: a membrane-based synthetic approach. Science 266, 1961–1966 (1994) . 17836514 10.1126/science.266.5193.1961

[b19] Li C., Bai H. & Shi G. Conducting polymer nanomaterials: electrosynthesis and applications. Chem. Soc. Rev. 38, 2397–2409 (2009) . 19623357 10.1039/b816681c

[b20] Zhang X. & Manohar S. K. Bulk synthesis of polypyrrole nanofibers by a seeding approach. J. Am. Chem. Soc. 126, 12714–12715 (2004) . 15469232 10.1021/ja046359v

[b21] Shi W., Liang P., Ge D., Wang J. & Zhang Q. Starch-assisted synthesis of polypyrrole nanowires by a simple electrochemical approach. Chem. Commun. 2414–2416 (2007) . 10.1039/b701592e 17844764

[b22] Curtis C. L., Ritchie J. E. & Sailor M. J. Fabrication of conducting polymer interconnects. Science 262, 2014–2016 (1993) . 17794966 10.1126/science.262.5142.2014

[b23] Fujii M., Arii K. & Yoshino K. Neuron-type polypyrrole device prepared by electrochemical polymerization method and its properties. Synth. Met. 71, 2223–2224 (1995) .

[b24] Bradley J.-C. . Creating electrical contacts between metal particles using directed electrochemical growth. Nature 389, 268–271 (1997) .

[b25] Moradi A., Emamgolizadeh A., Omrani A. & Rostami A. A. Electropolymerization and characterization of 3,4-ethylenedioxy thiophene on glassy carbon electrode and study of ions transport of the polymer during redox process. J. Appl. Polym. Sci. 125, 2407–2416 (2012) .

[b26] Melato A. I., Mendonca M. H. & Abrantes L. M. Effect of electropolymerisation conditions on the electrochemical, morphological and structural properties of PEDOTh films. J. Solid State Electrochem. 13, 417–426 (2009) .

[b27] Wolfs M., Darmanin T. & Guittard F. Versatile superhydrophobic surfaces from a bioinspired approach. Macromolecules 44, 9286–9294 (2011) .

