# Supplementary material for: Electropolymerization on wireless electrodes towards conducting polymer microfibre networks
Source: Nat Commun. 2016 Jan 25;7:10404. doi: 10.1038/ncomms10404 (PMC4737731; doi:10.1038/ncomms10404)
Supplement: Supplementary Information — Supplementary Figures 1-13, Supplementary Methods and Supplementary References [file ncomms10404-s1.pdf]

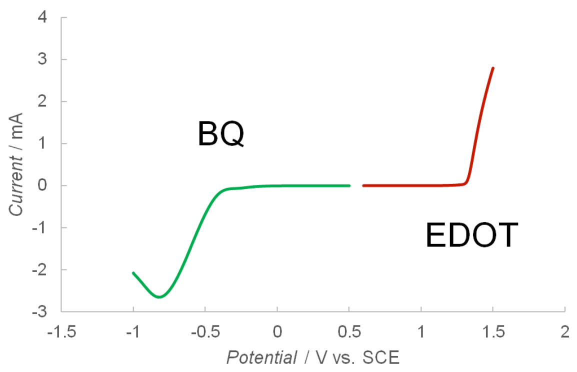

Supplementary Figure 1. Linear sweep voltammograms of EDOT (10 mM) and BQ (10 mM) measured independently in 0.1 M Bu_4NClO_4/MeCN using ITO working electrode (10 mm × 10 mm) at a scan rate of 100 mV/s.

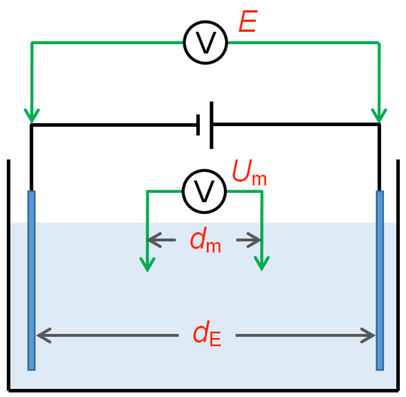

Supplementary Figure 2. Cell configuration and definition of the parameters for the equations.

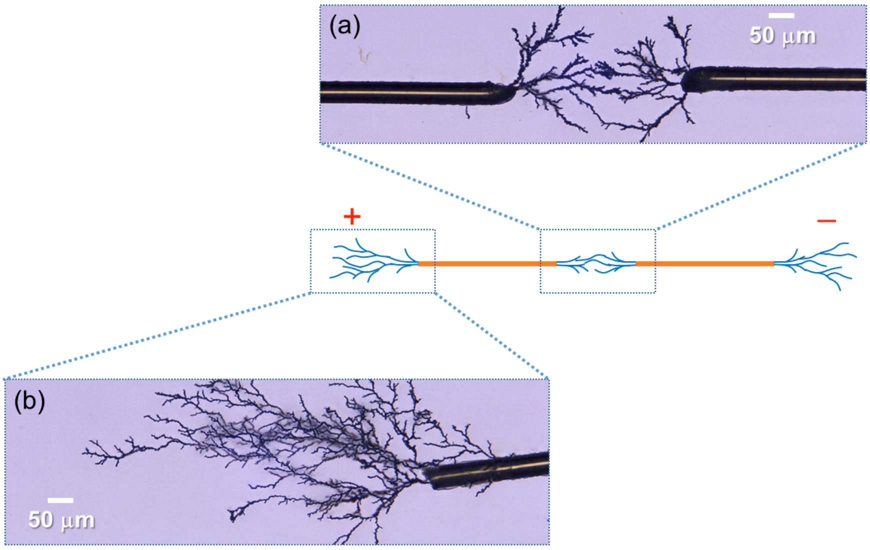

Supplementary Figure 3. Optical microscope images of PEDOT fibers connecting two Au wires (0.3 mm gap): (a) a gap that was bridged in 20 sec following which the propagation was stopped, and (b) the opposite end of the Au wire, demonstrating the growth of PEDOT fibers over an additional 120 sec.

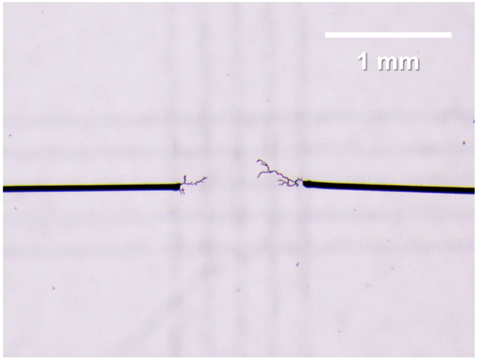

Supplementary Figure 4. Optical microscope image of PEDOT fibers propagated from the Au wires (ΔV_{BPE} = 5.8 V, 90 sec).

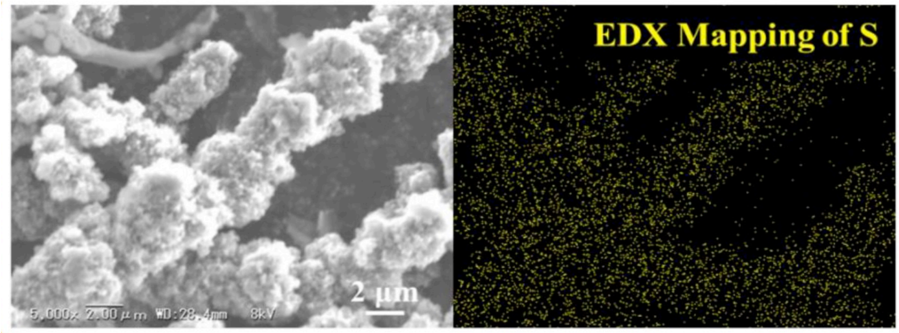

Supplementary Figure 5. EDX mapping of sulfur atoms in the corresponding SEM image of PEDOT fibers shown in Figure 2c.

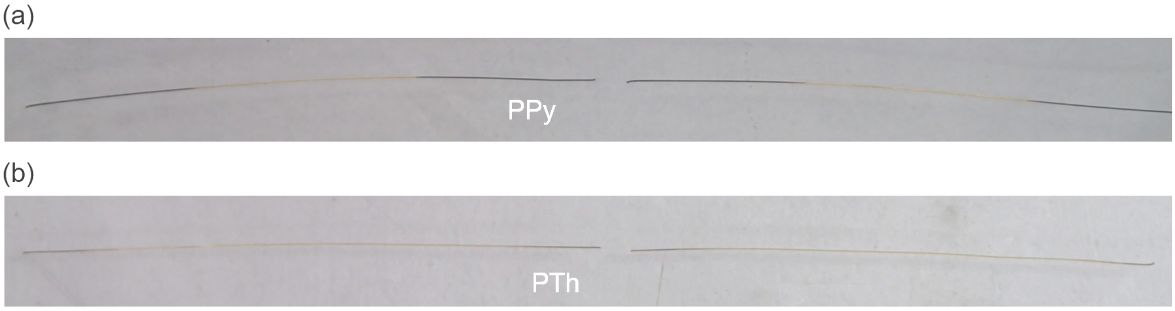

Supplementary Figure 6. Optical microscope images of Au wires (20 mm length) covered with (a) polypyrrole (PPy) and (b) polythiophene (PTh) after AC-bipolar electropolymerization of the corresponding monomers.

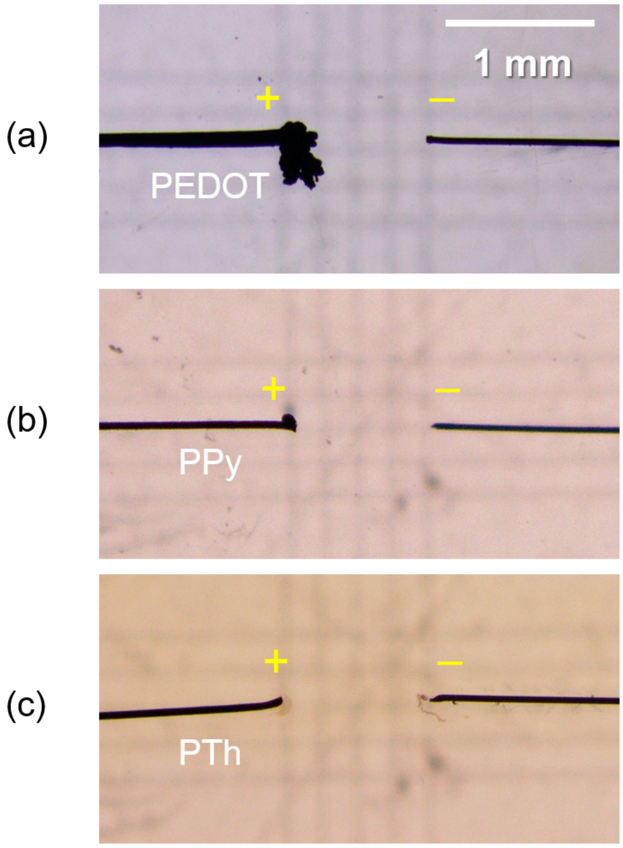

Supplementary Figure 7. Optical microscope images of Au wires after DC-bipolar electropolymerization of (a) EDOT, (b) pyrrole and (c) thiophene for 3 min.

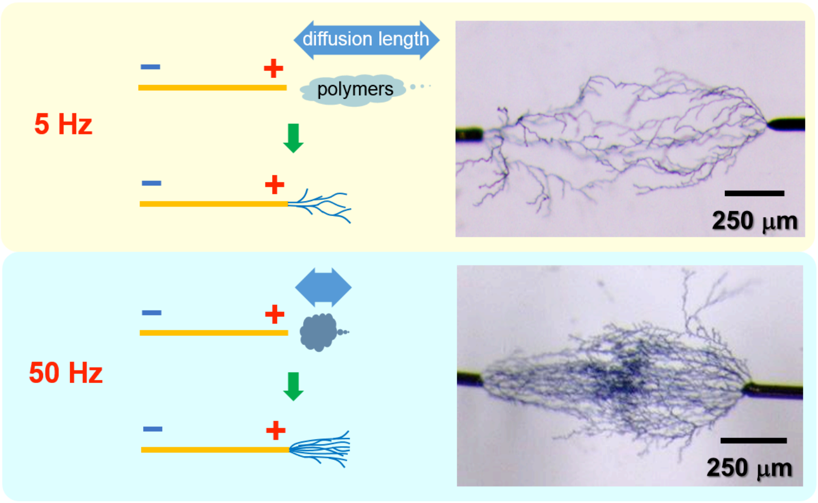

Supplementary Figure 8. Optical microscope images of the connected PEDOT fibers obtained with different frequencies.

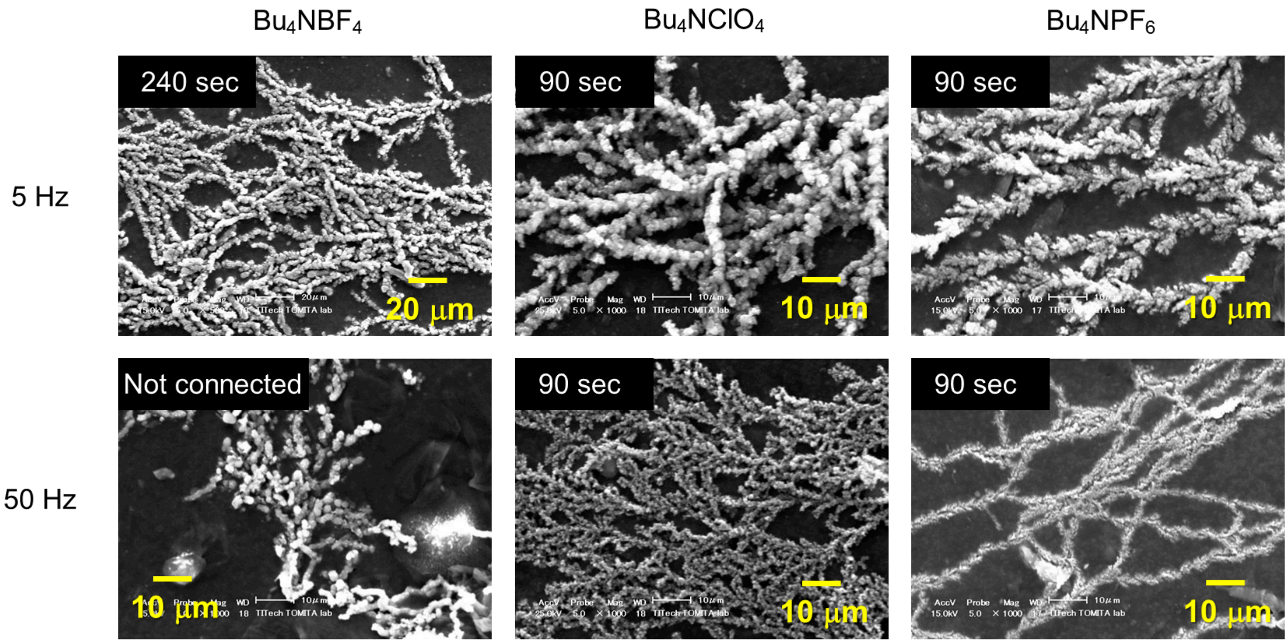

Supplementary Figure 9. SEM images of PEDOT fibers obtained under different frequencies and with various supporting salts. The time span for connecting 1 mm gap between the BPEs is inserted for each entry.

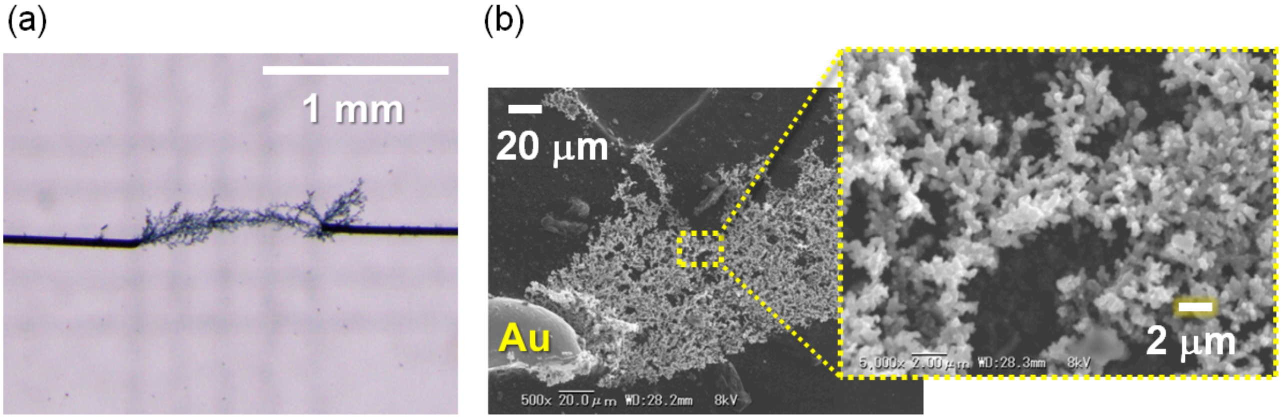

Supplementary Figure 10. (a) Optical microscope and (b) SEM images of the PEDOT fibers prepared in Bu_4NClO_4/CH_2Cl_2.

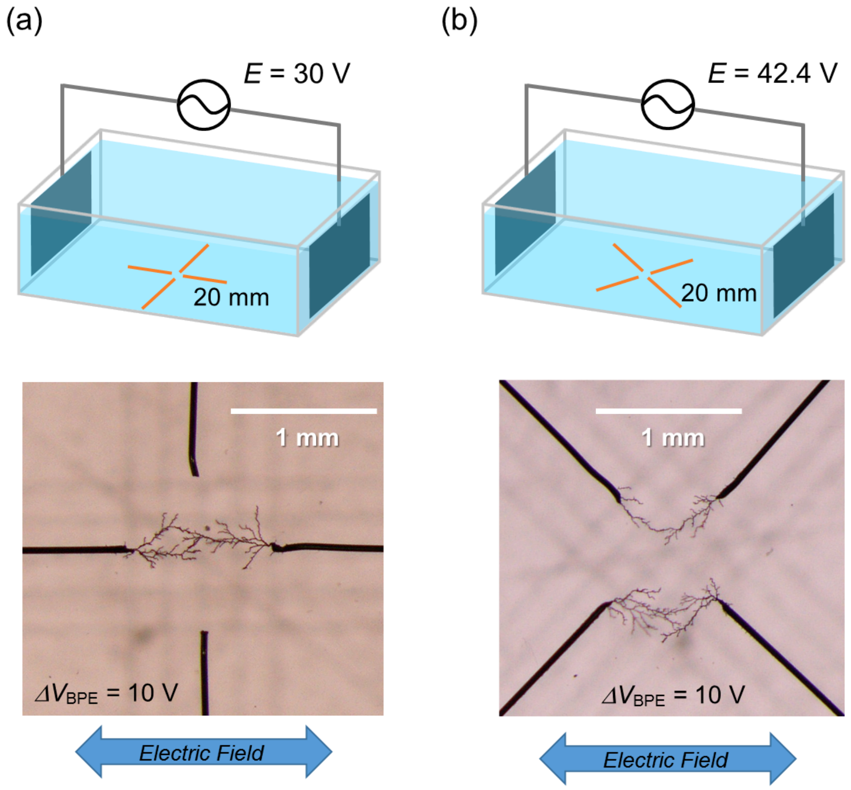

Supplementary Figure 11. (a) Schematic illustration of the setup for network formation at an intersection of BPEs (top) and the optical microscope image of the PEDOT networks after the electrolysis (ΔV_{BPE} = 10 V, 5 Hz, 90 sec) (bottom). (b) Similar illustration and microscope image of the different configuration of BPEs (ΔV_{BPE} = 10 V, 5 Hz, 60 sec).

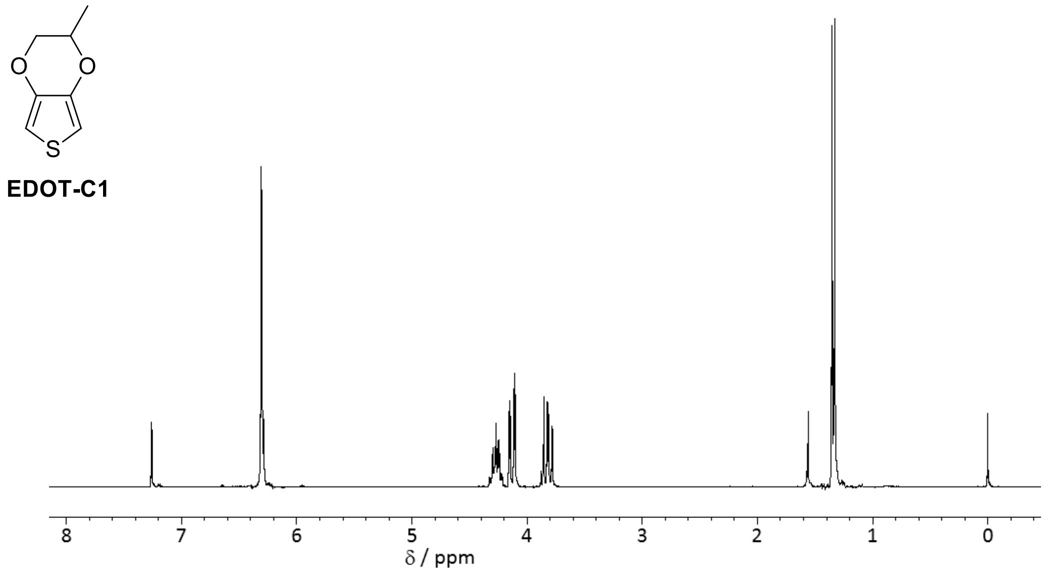

Supplementary Figure 12. ^{1}H NMR spectrum of EDOT-C1 in $CDCl_3$.

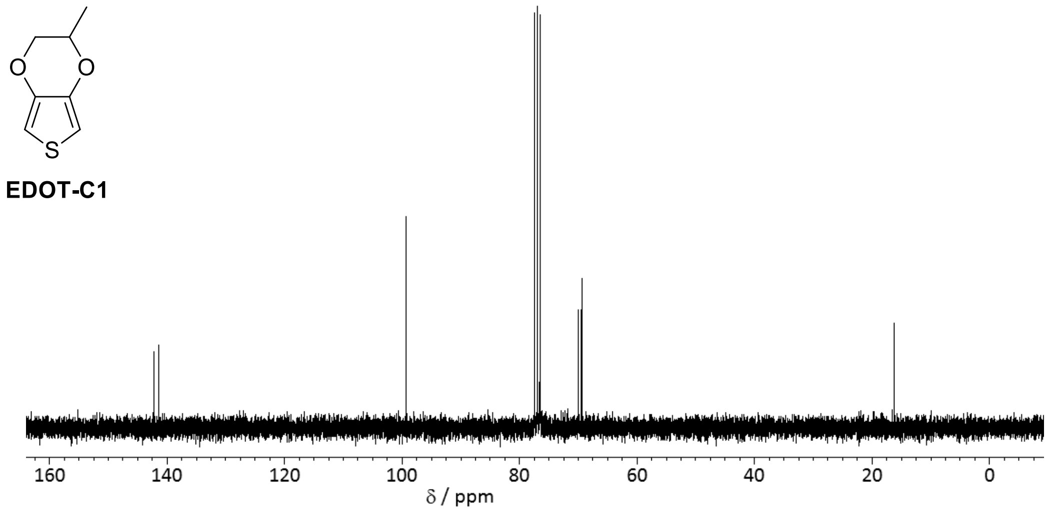

Supplementary Figure 13. ^{13}C NMR spectrum of EDOT-C1 in $CDCl_3$.

Supplementary methods

Cell configuration

The glass cell shown in Figure 2a, equipped with Pt driving electrodes (20 mm × 20 mm, distance: 60 mm), was filled with an electrolytic solution. Two Au wires (ϕ = 50 μm, 20 mm) were placed 1 mm apart from one another between the driving electrodes.

Estimation of ΔV_{min}

The minimum applied voltage required to induce BPE (ΔV_{min}) was estimated from the difference between the oxidation and reduction potentials of the corresponding species, E_{ox} and E_{red}, (Supplementary Eq. 1), as previously reported[1].

$$\Delta V_{\mathrm{min}} = |E_{\mathrm{ox}} - E_{\mathrm{red}}| \qquad (1)$$

In our system, the oxidation of EDOT and the reduction of BQ were assumed to occur simultaneously on the BPE. Using the onset potentials of these half reactions (Supplementary Figure 1), ΔV_{min} was estimated using Supplementary Eq. 2.

$$\Delta V_{\mathrm{min}} = |E_{\mathrm{ox}} - E_{\mathrm{red}}| = |1.33\ \mathrm{V} - (-0.49\ \mathrm{V})| = 1.82\ \mathrm{V} \qquad (2)$$

Estimation of cell factors and ΔV_{BPE}

In order to estimate a cell factor, i.e., the electric field transmission efficiency θ, a ratio of electric field inside the cell ($\varepsilon_{\mathrm{eff}}$) and applied electric field between driving electrodes (ε) is defined

as follows.[1,2]

$$\text{[illegible]} = \frac{\text{[illegible]}_{\mathrm{eff}}}{\text{[illegible]}} \qquad (3)$$

It follows that

$$\text{[illegible]} = \frac{U_m\, d_E}{d_m\, E} \qquad (4)$$

and

$$U_m = \text{[illegible]}\ \frac{d_m\, E}{d_E} \qquad (5)$$

where U_m is the potential difference measured between two microelectrodes with distance of d_m, E is the applied voltage between the driving electrodes set with distance of d_E (Supplementary Figure 2). Based on Eq. 5 a value of θ can be estimated from slope of plots of U_m as a function of Ed_m/d_E. Thus a potential applied between each bipolar electrode (ΔV_{BPE}) can be estimated as follows:

$$\text{[illegible]}V_{BPE} = \text{[illegible]}\ \frac{d_{BPE}\, E}{d_E} \qquad (6)$$

where d_{BPE} is the length of bipolar electrode. The value of ΔV_{BPE} was stable for the time scale of the electrolysis.

Supplementary References

1. Loget, G., Roche, J. & Kuhn, A. True bulk synthesis of Janus objects by bipolar electrochemistry. *Adv. Mater.* **24**, 5111–5116 (2012).
2. Koizumi, Y., Shida, N., Tomita, I. & Inagi, S. Bifunctional modification of conductive particles by iterative bipolar electrodeposition of metals. *Chem. Lett.* **43**, 1245–1247 (2014).
